# Expression of Cytokine-Coding Genes *BMP8B*, *LEFTY1* and *INSL5* Could Distinguish between Ulcerative Colitis and Crohn’s Disease

**DOI:** 10.3390/genes12101477

**Published:** 2021-09-23

**Authors:** Daša Jevšinek Skok, Nina Hauptman, Miha Jerala, Nina Zidar

**Affiliations:** 1 Institute of Pathology, Faculty of Medicine, University of Ljubljana, Korytkova 2, SI-1000 Ljubljana, Slovenia; dasa.jevsinekskok@kis.si (D.J.S.); nina.hauptman@mf.uni-lj.si (N.H.); miha.jerala@mf.uni-lj.si (M.J.); 2 Agricultural Institute of Slovenia, Hacquetova Ulica 17, SI-1000 Ljubljana, Slovenia

**Keywords:** inflammatory bowel disease, ulcerative colitis, Crohn’s disease, inflammatory cytokines, expression, bioinformatics approach, experimental validation

## Abstract

Ulcerative colitis (UC) and Crohn’s disease (CD) are characterized by an imbalance between pro-inflammatory and anti-inflammatory cytokines, interfering with the resolution of inflammation. Due to the crucial role of cytokines, new insights into their profiles in UC and CD would help to improve our understanding of pathogenesis and enable the development of new treatment modalities. We provide an expression profile of cytokines in UC and CD, using bioinformatics approach, and experimental validation of expression of the selected genes. We retrieved data and analyzed the cytokine gene expression profiles of UC and CD. From ten genes with inverse expression, common to CD and UC, *BMP8B*, *LEFTY1* and *INSL5* were selected for gene expression experimental validation. Experimentally, *BMP8B* and *INSL5* were down-regulated in both CD and UC but followed the bioinformatics trend. The expression of genes *LEFTY1* and *BMP8B* was statistically significant when comparing UC and CD in colon and the expression of gene *LEFTY1* showed statistical significance when CD in ileum and colon were compared. Using the bioinformatics approach and experimental validation, we found differences in expression profiles between UC and CD for *INSL5*, *LEFTY1* and *BMP8B*. These three promising candidate genes need to be further explored at different levels, such as DNA methylation and protein expression, to provide more evidence on their potential diagnostic role in CD and UC.

## 1. Introduction

Despite significant progress in our understanding of both major forms of inflammatory bowel diseases (IBD), ulcerative colitis (UC) and Crohn’s disease (CD), many questions remain unresolved, such as the exact pathogenesis and the reason for different macroscopic, microscopic, and clinical features of UC and CD [1,2]. Both UC and CD develop as a result of an interaction between genetic and environmental factors, which promotes an excessive response of gut-associated immune system against the gut microbiota. The resulting chronic inflammation is mediated by cytokines, which play a crucial role in intestinal inflammation and the associated clinical symptoms but may also regulate extra-intestinal disease manifestations and systemic effects of IBD. The situation is even more complex as cytokines may play not only a pro-inflammatory role, but also a protective, regulatory role. It is now believed that in IBD, the imbalance between pro-inflammatory and anti-inflammatory cytokines interferes with the resolution of inflammation and leads to chronic disease with progressive tissue destruction [3].

Despite some similarities between CD and UC, the effector pathways including the cytokine profiles through which tissue injury is mediated are distinct for each disease. Such differences may be responsible not only for several clinicopathological dissimilarities that clearly separate UC from CD, but also for inter-individual variations, including disparate response to therapeutic agents [1,4]. Clearly, new insights into the cytokine profiles in UC and CD would not only help to improve our understanding of the pathogenesis of IBD, but most importantly would enable the development of new treatment modalities. The aim of the present study is to provide an expression profile of cytokines in UC and CD, using bioinformatics approach, and experimental validation of the selected cytokine coding genes.

## 2. Materials and Methods

### 2.1. Microarray Data

Four projects, GSE13367 [5], GSE48958 [6], GSE53306 [7] and GSE65114, with samples of gene expression profiles of UC and three, GSE24287 [8], GSE66207 [9] and GSE100833 [10], with samples of gene expression profile of CD were obtained from the public functional genomics data repository—Gene Expression Omnibus database (GEO, http://www.ncbi.nlm.nih.gov/geo, accessed on 25 January 2019) of The National Center for Biotechnology Information (NCBI). In case of UC, only active UC samples were selected for our analysis and are here denoted as UC samples. A total of 47 UC, 226 CD and 247 normal tissue samples were included in this study (Table 1).

### 2.2. Data Processing

For all projects, the original data files were downloaded and further normalized in R language (https://www.r-project.org/, accessed on 25 January 2019). For projects based on Affymetrix arrays (GSE13367, GSE48958, GSE65114 and GSE100833) package “affy” [11] was used to convert CEL files into expression data using the robust multichip average function, which performs background correction and normalization in one step. For projects based on other arrays (GSE53306, GSE24287), package “limma” [12] was used to perform background correction and normalization between arrays. After data normalization, a gene filter was used to remove probes that had an intensity of less than 100 in more than 20% of samples in each project. For project GSE66207, the matrix of already normalized counts was downloaded and used as was.

Differentially expressed genes (DEG) were identified on probe level using “limma” package in R for each individual project. We constructed two contrast matrices (UC compared to normal and CD compared to normal) for each GEO project. The cut-off conditions were set to adjusted *p*-value < 0.05 and logFC > 0.5.

The list of 456 cytokines was obtained from the ImmPort portal (http://www.immport.org/immport-open/public/reference/genelists, accessed on 18 October 2019) and overlapped with the genes from analyzed GEO projects (Table S1).

### 2.3. Functional Analysis

For functional analysis and construction of protein-protein interactions (PPI) network, the Search Tool for the Retrieval of Interacting Genes (STRING) database [13] and GeneTrail [14] were employed. In this study, we constructed PPI networks of DEGs for both comparisons. The PPI network was visualized under the cut-off of interaction score of 0.4.

Besides constructing PPI networks, STRING database enables functional and pathway enrichment analysis. STRING offers integrative tools for uncovering the biological meaning behind large sets of genes. Gene ontology (GO) analysis including biological process (6168 processes), molecular function (758) and cellular component (1550), as well as Kyoto Encyclopedia of Genes and Genomes (KEGG) Pathways [15], were conducted for selected DEGs with GeneTrail2 and STRING. Pathway analyses were conducted by GeneTrail2, where given dataset is compared to 310 pathways according to BioCarta, 280 from KEGG Pathways, 1300 from Reactome and 106 from PharmGKB database. In addition, 853 drug targets from DrugBank and Phenotype data (704) from NIA-Phenotypes were included in our study. The statistical significance threshold was set at *p* < 0.05. Over-representation analysis (ORA) as an enrichment algorithm with FDR adjustment method was used for determining the number of statistically significant categories [14].

### 2.4. Patients and Tissue Samples

Our study included endoscopic biopsies collected during routine ileocolonoscopies from 31 patients with UC and 25 patients with CD. We collected 27 samples of CD, 35 samples of UC, and 22 samples of the normal mucosa. All of the fresh tissue samples were fixed in formalin and embedded in paraffin. Paraffin blocks were cut into sections and stained with hematoxylin and eosin for histopathological assessment, which was performed by experienced pathologists. Patients’ characteristics are presented in Table 2.

### 2.5. RNA Isolation and Reverse Transcription

When available, ten 10 µm thick sections were cut from each paraffin block for RNA isolation, which was performed with All Prep DNA/RNA FFPE Kit according to the manufacturer’s instructions (Qiagen, Hilden, Germany; cat. no. 80234). Concentration of RNA was measured on NanoDrop-1000 (Thermo Fisher Scientific, Waltham, MA, USA). Reverse transcription of RNA was conducted using OneTaq RT-PCR Kit (New England Biolabs, Ipswich, Massachusetts, USA; cat. no. E5310S) according to the manufacturer’s instructions, with half reaction volume. Each reaction contained 3 µL of total RNA with concentrations of 125 ng/µL, 1 µL 60 µM Random Primer Mix, 5 µL 2X M-MuLV Reaction Mix and 1 µL 10X M-MuLV Enzyme Mix. After the RNA and Random Primer Mix were mixed and incubated for 5 min at 70 °C, Reaction and Enzyme Mix were added and whole reactions was further incubated for 5 min at 25 °C, 60 min at 42 °C and 4 min at 80 °C.

### 2.6. Preamplification and Quantitative Real Time PCR

After the reverse transcription, cDNA was pre-amplified using TaqMan PreAmp MasterMix (Thermo Fisher Scientific, cat. no. 4488593), according to manufacturer’s instructions. Briefly, 10 µL reactions contained 2.5 µL of cDNA, 2.5 µL of 0.2X pooled TaqMan GeneExpression Assays (Thermo Fisher Scientific, cat. no. 4331182) (Table 3) and 5 µL 2X TaqMan PreAmp MasterMix. Pooled TaqMan GeneExpression Assays were diluted to 0.2X using TE buffer solution (Sigma Aldrich, Burlington, MA, USA; cat. no. 93302-500ML). Preamplification reaction conditions were: 10 min at 95 °C and 10 cycles of 15 s at 95 °C and 4 min at 60 °C. Preamplification reactions were diluted 15-fold to a final volume of 150 µL. Each 10 µL qPCR reaction contained 4.5 µL of diluted preamplified cDNA, 5 µL 2X FastStart Essential DNA Probe Master (Roche, Basel, Switzerland; cat. no. 06924492001) and 0.5 µL TaqMan GeneExpression Assay (Table 3). All qPCR reactions were performed in duplicates on Rotor-Gene Q (Qiagen). Cycling conditions were: 2 min at 50 °C, 10 min at 95 °C and 40 cycles of 15 s at 95 °C and 1 min at 60 °C.

### 2.7. Statistical Analysis of qPCR

To calculate the relative gene expression, Cqs were obtained as average of duplicates for each sample. To obtain ∆Cq, a geometric average of Cqs of reference genes (*IPO8* and *B2M*) was deducted from gene of interest (*BMP8B*, *LEFTY1* and *INSL5*). All statistical analyses were performed using SPSS analytical software ver. 24, (SPSS Inc., Chicago, IL, USA) with cut-off *p* ≤ 0.05. For group comparison, a statistically significant difference was calculated using ∆Cq and Mann–Whitney test.

## 3. Results

### 3.1. Differentially Expressed Genes in Ulcerative Colitis and Crohn’s Disease

Data from each microarray were separately analyzed to obtain differentially expressed genes (DEGs) for each comparison, UC compared to normal, and CD compared to normal. The resulting gene list was overlapped with 456 cytokines and used for further analysis. We identified 201 statistically significant differentially expressed cytokine-coding genes in UC and 36 in CD tissue samples (Table S2). There were 28 genes differentially expressed common to both UC and CD when compared to normal. In addition, we identified unique DEGs in each group, 173 in UC and eight in CD.

**Figure 1:**
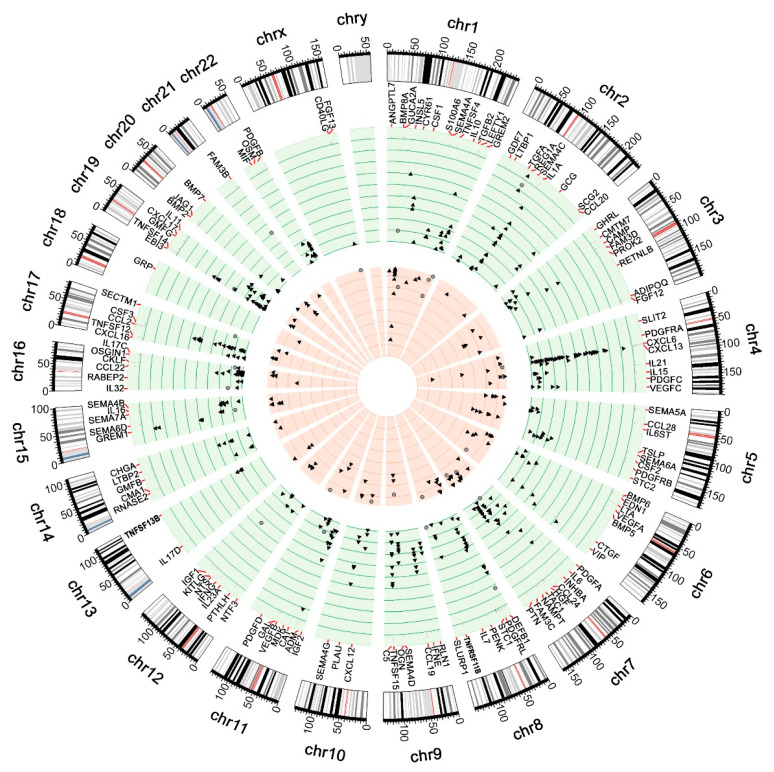
Differential expression profile of ulcerative colitis (triangles) and Crohn’s disease (circles). Green background, up-regulated cytokines; red background, down-regulated cytokines; green axes, logFC between 0.5 and 5; red axes, logFC between −0.5 and −4.

Most of the genes were up-regulated in both groups (Figure 1), although we observed some differences between gene expression profiles among the groups. Among 28 common DEGs, we focused on 10 DEGs where all probes exhibit inverse regulation between CD and UC groups. From largest to smallest difference between groups, these DEGs are presented in Table 4.

### 3.2. Functional Enrichment Analysis

To obtain a better understanding of cytokine functions in UC and CD, we performed a functional enrichment analysis for each group and their union (Table 5). The analysis of gene ontology (GO), pathways, drug targets and associations with phenotypes was performed for 201 differentially expressed genes in UC and 36 in CD, and all the results for all terms can be viewed in Table S3.

#### 3.2.1. Biological Processes

Our CD and UC sets of differentially expressed cytokine-coding genes were compared with GeneTrail2 GO knowledgebase, which contains 6168 biological processes, 758 cellular components and 1550 molecular functions. We found 1322 significant biological processes in UC and 100 within the CD DEGs dataset. Overall, 1323 biological processes were significant in at least one dataset, with the most significant biological processes in both datasets: positive regulation of behavior, positive regulation of chemotaxis, regulation of leukocyte migration.

The *INSL5* gene is included in three biological processes: feeding behavior, positive regulation of behavior and regulation of feeding behavior. The *LEFTY1* gene is included in the following biological processes: heart morphogenesis and transforming growth factor-β receptor signaling pathway. Biological processes for gene *BMP8B* include: BMP signaling pathway, cartilage development, connective tissue development and response to BMP and cellular response to BMP stimulus. In addition, genes *LEFTY1* and *BMP8B* share six biological processes, namely: pathway restricted SMAD protein phosphorylation, positive regulation of pathway restricted SMAD protein phosphorylation, positive regulation of transmembrane receptor protein serine threonine kinase signaling pathway, regulation of pathway restricted SMAD protein phosphorylation, positive regulation of transmembrane receptor protein serine threonine kinase signaling pathway and SMAD protein signal transduction.

#### 3.2.2. Cellular Components

A total of four cellular components were found to be significant, all of them in the UC dataset: secretory granule lumen, platelet α-granule lumen, platelet α-granule and Golgi lumen. In the CD dataset, there was no significant term for cellular component.

#### 3.2.3. Molecular Functions

A total of 41 molecular functions were significant in at least one of the compared datasets; 14 of them were found as significant in both datasets. The top five molecular functions significant in both datasets are: chemokine activity, chemokine receptor binding, growth factor receptor binding, heparin binding and chemoattractant activity.

The *INSL5* gene is not included in any molecular function from the list, while the *LEFTY1* gene is included in transforming growth factor-β receptor binding molecular function. The *BMP8B* is included in four molecular functions, named BMP receptor binding, receptor serine threonine kinase binding, transforming growth factor-β receptor binding and transmembrane receptor protein serine threonine kinase binding.

#### 3.2.4. Pathway Analysis

**Figure 2:**
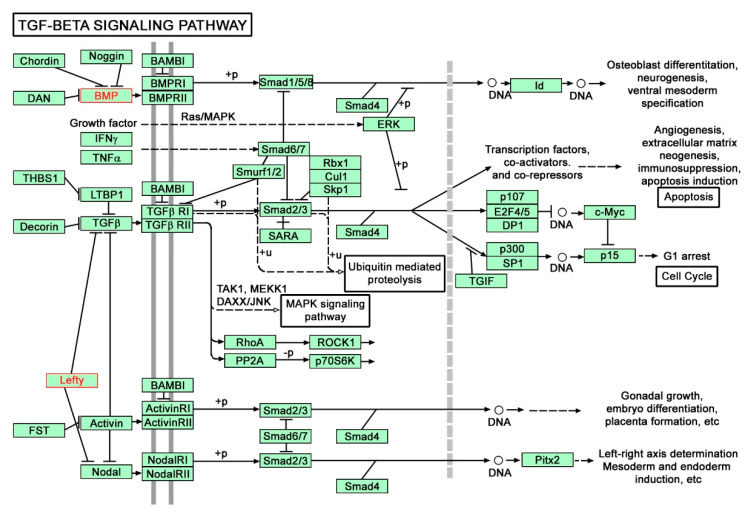
Genes mapping to KEGG pathway for TGF-β signaling pathway (hsa04350) [5] with included *LEFTY1* and *BMP8B* genes (red labels).

We performed pathway analysis according to the three well-known databases: Biocarta, KEGG and Reactome. We found 22 significant molecular pathways according to Biocarta, 61 according to KEGG and 47 according to Reactome in at least one group of differentially expressed cytokine-coding genes (Supplementary Table S2). Most of the pathways are associated with cytokines. The top three pathways from each database include: cytokines and inflammatory response, erythrocyte differentiation pathway and cytokine network (Biocarta), chemokine signaling pathway, cytokine-cytokine receptor interaction and TGF-β signaling pathway (KEGG), chemokine receptors bind chemokines, Gα (i) signaling events and peptide ligand-binding receptors (Reactome). The *INSL5* gene is included in Gα (i) signaling events and relaxin receptors pathway according to Reactome database. The *LEFTY1* and *BMP8B* genes are included in KEGG pathway TGF-β signaling (Figure 2). In addition, gene *BMP8B* is included in Hippo signaling pathway (figure not shown).

#### 3.2.5. Phenotypes

There were 129 significant phenotypes in at least one dataset (UC, CD). The GeneTrail2 software was unable to find any phenotypes based on CD dataset due to a small number of genes differentially expressed in CD tissue. The top phenotypes significant in UC dataset were asthma, disease progression, hepatitis B, acute disease, hypertension, lupus erythematosus and inflammation. Inflammation phenotype contains ten genes from our lists: *CSF3*, *IL10*, *IL1A*, *IL1RN*, *IL6*, *LTA*, *MIF*, *NAMPT*, *SPP1* and *TNF*. Comparing our datasets with available phenotype data for acute disease, we found 10 genes in common: *CAT*, *CXCL12*, *IFNG*, *IL10*, *IL1A*, *IL1B*, *IL1RN*, *IL6*, *MIF*, *TNF* and *VEGFA*.

In addition, Crohn Disease and Colitis were also found to be significant phenotypes based on UC datasets. The phenotype named Colitis contains seven DEGs from our lists: *IL10*, *IL11*, *IL1B*, *IL1RN*, *IL6*, *MIF* and *TNF*. Within the phenotype named Crohn Disease, nine genes from UC datasets were included: *CCL2*, *IL10*, *IL16*, *IL1B*, *IL1RN*, *IL23A*, *IL6*, *MIF* and *TNF*.

#### 3.2.6. Protein-Protein Interactions Network

**Figure 3:**
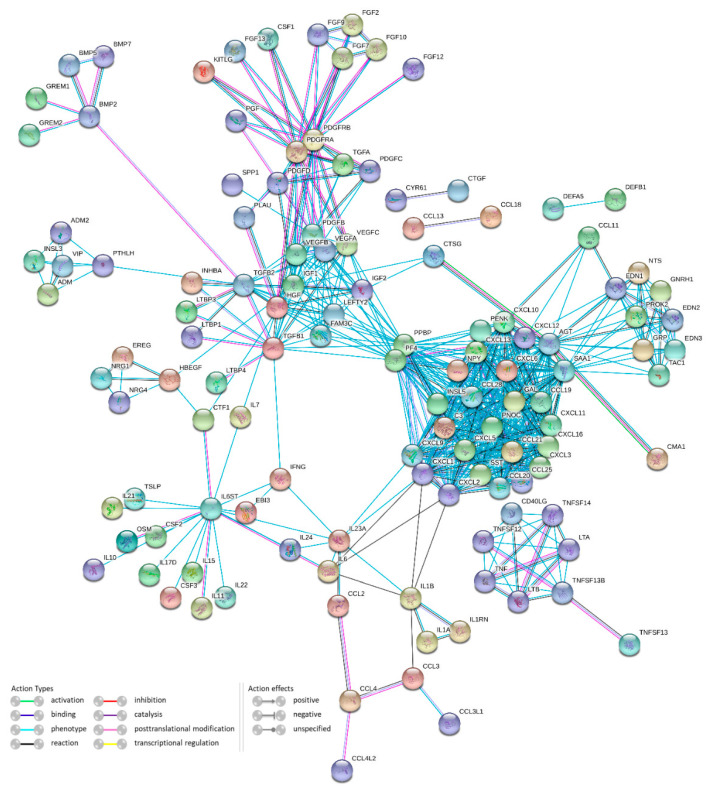
Protein-protein interaction network with proteins coded by DEGs in ulcerative colitis and/or Crohn’s disease.

The STRING database was used to study the proteome organization of differences between UC and CD and to construct protein-protein interaction (PPI) networks. Figure 3 represents a network of genes differentially expressed in our analysis. In the whole network, the top five hubs are AGT (40 interactions), PF4 and PPBP (39 interactions), SAA1 (37 interactions) and CXCL1 (31 interactions).

### 3.3. Experimental Results

To test our bioinformatics analysis, we selected three genes among ten that expressed inverse regulation among CD and UC groups, for further experimental validation with quantitative polymerase chain reaction (qPCR). We selected *INSL5*, which had the largest difference in logFC among UC and CD (ΔlogFC = 4.49), *BMP8B* with the smallest difference in logFC among CD and UC (ΔlogFC = 1.37), and *LEFTY1*, where the difference between UC and CD was somewhere in the middle (ΔlogFC = 2.30) (Table 1).

**Figure 4:**
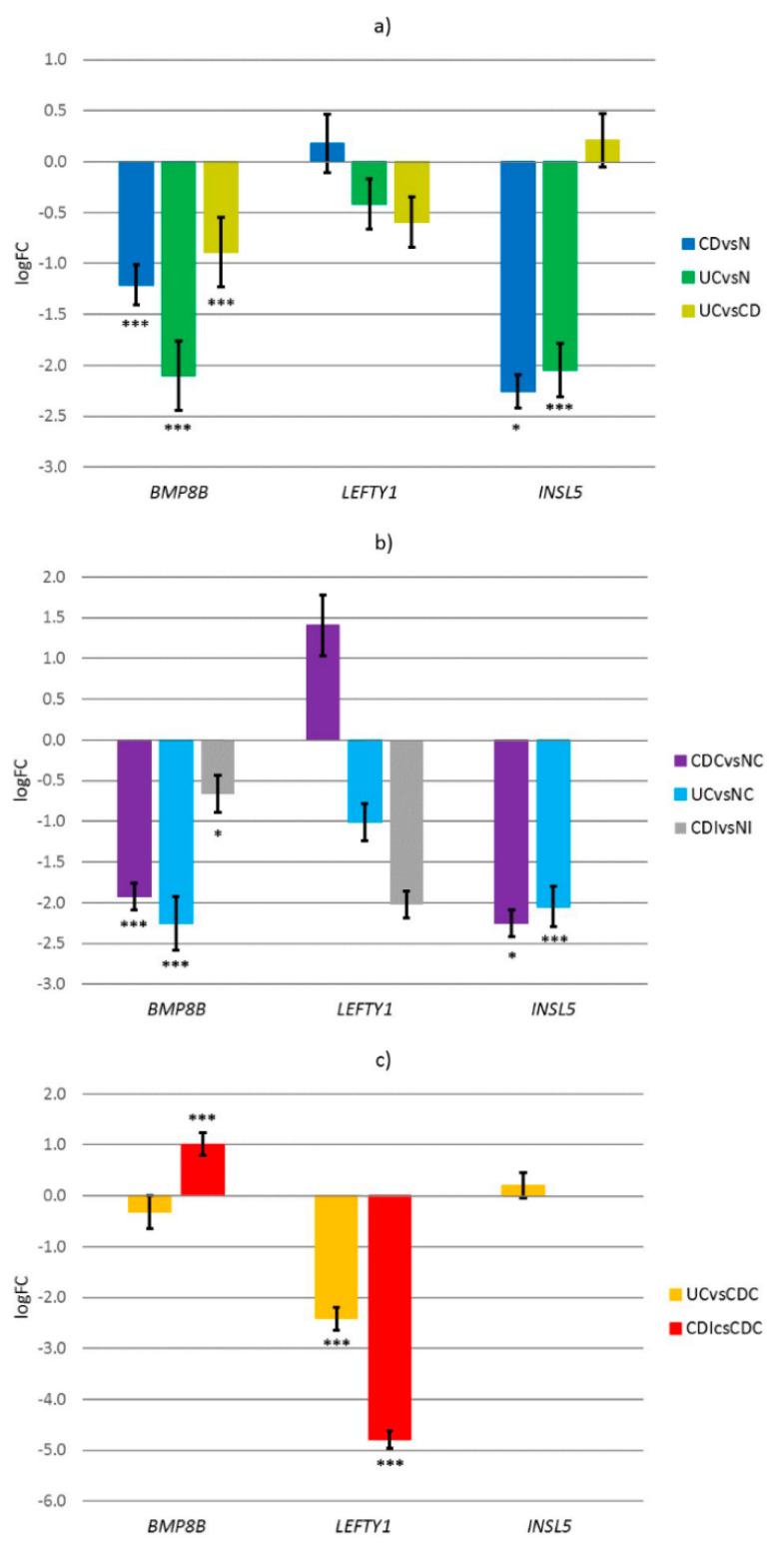
Expression of *BMP8B*, *LEFTY1* and *INSL5* of (**a**) Crohn’s disease compared to normal (blue), ulcerative colitis compared to normal (green) and ulcerative colitis compared to Crohn’s disease (yellow), (**b**) Crohn’s disease in colon compared to normal colon (violet), ulcerative colitis in colon compared to normal colon (light blue) and Crohn’s disease in ileum compared to normal ileum (gray), (**c**) ulcerative colitis in colon compared to Crohn’s disease in colon (orange), Crohn’s disease in ileum compared to Crohn’s disease in colon (red); lines represent error; *** *p* < 0.001, * *p* < 0.05; CD, Crohn’s disease; UC, ulcerative colitis; N, normal mucosa; CDC, Crohn’s disease in colon; UC, ulcerative colitis in colon; NC, normal colon mucosa; CDI, Crohn’s disease in ileum; NI, normal ileum mucosa.

Our experimental results show that *BMP8B* and *INSL5* are both down-regulated in both CD compared to normal (*BMP8B* logFC = −1.21, *p* < 0.001; *INSL5* logFC = −2.26, *p* = 0.048), and UC compared to normal (*BMP8B* logFC = −1.74, *p* < 0.001; *INSL5* logFC = −2.05, *p* = 0.008), while *LEFTY1* expressed low difference in expression (CD logFC = 0.18, UC logFC = −0.15), which was not significant. The difference in expression between CD and UC was significant in case of *BMP8B* (logFC = −0.53, *p* = 0.010), while expression in *LEFTY1* and *INSL5* was not significant for this comparison (Figure 4a).

When dividing the samples based on location, either colon or ileum, further comparisons were made, e.g., CD in colon (CDC) compared to normal colon (NC), UC in colon (UC) compared to NC and CD in ileum (CDI) compared to normal ileum (NI). In the colon, the expression of *BMP8B* and *INSL5* was in both comparisons CDC compared to NC (*BMP8B* logFC = −1.93, *p* < 0.001; *INSL5* logFC = −2.26, *p* = 0.048) and UC compared to NC (*BMP8B* logFC = −2.25, *p* < 0.001; *INSL5* logFC = −2.05, *p* = 0.008) down-regulated, while *LEFTY1* was up-regulated in CDC compared to NC (logFC = 1.40) and down-regulated in UC compared to NC (logFC = −1.01) but in neither case the expression was significant. In the ileum, *INSL5* was not expressed in any group, not even in the NI. We observed the decreased expression of both *BMP8B* (logFC = −0.67, *p* = 0.041) and *LEFTY1* (logFC = −2.02) in CDI compared to NI (Figure 4b).

Further comparisons were made between the different disease states in different locations. We observed a difference between the expression of UC compared to CDC in gene *LEFTY1* (logFC = −2.42, *p* < 0.001), while the expressions of *BMP8B* (logFC = −0.32) and *INSL5* (logFC = 0.21) were not statistically significant. In ileum, *INSL5* was not expressed, *BMP8B* expression increased (logFC = 1.01, *p* = 0.002) and *LEFTY1* expression decreased (logFC = −4.79, *p* < 0.001) when comparing CD in different locations, CDI to CDC (Figure 4c).

## 4. Discussion

In this study, we analyzed the gene expression profiles of UC and CD according to seven GEO projects. We identified 201 statistically significant differentially expressed cytokine-coding genes in UC and 36 in CD tissue samples. We identified 28 genes common to UC and CD, and 10 genes which exhibit inverse regulation in UC and CD. Among those 10 genes, 3 of them *FGF2*, *NTS* and *VIP*, were previously associated with IBD [16,17,18,19,20,21,22].

According to the difference in expression, we selected three genes for further experimental validation, *INSL5* with the highest difference in expression between UC and CD, *BMP8B* with the smallest difference in expression, and *LEFTY1*, which was the fifth gene with the highest difference in expression among UC and CD groups.

According to our bioinformatics analysis, *INSL5* was up-regulated in UC and down-regulated in CD. Our experiment showed that *INSL5* was down-regulated in both CD and UC groups, more in CD than in UC, confirming the trend of expression from bioinformatics study. Furthermore, we discovered that *INSL5* was not expressed in the ileum, not even in the normal ileum.

*INSL5* belongs to the insulin family, which includes insulin, insulin-like growth factors, relaxin and the insulin-like (INSL) peptides [23,24]. The insulin gene superfamily hormones regulate cell growth, metabolism, and tissue-specific functions [25]. The *INSL5* gene is expressed in various tissues, including the gastrointestinal tract [24]. However, reports on the presence of both *INSL5* and its receptor *RXFP4* in multiple peripheral tissues including the colon, placenta, heart, spleen, brain, prostate, kidney, bone marrow and liver implicate the involvement of this ligand receptor system in a broad spectrum of biological functions [24]. Increased levels of enteroendocrine cells (EECs) have been reported in the ileum from patients with CD [26,27,28] as well as in animal models of colitis [29,30,31]. Thanasupawat et al. revealed differentially expressed *INSL5* in EECs located within RXFP4 immuno-positive colonocytes [32].

The second gene we selected was *LEFTY1*, which was up-regulated in UC and down-regulated in CD, according to our bioinformatics analysis. The experiment showed a small deregulation in both CD and UC groups compared to the normal, but the difference was not significant. Interestingly, *LEFTY1* showed significant deregulation in expression when the disease states were compared to each other. It was down-regulated when UC was compared to CD in the colon and down-regulated when CD in the ileum was compared to CD in the colon. The expression of *LEFTY1* could be used to distinguish between UC and CD in the colon and between CD in the colon or ileum.

The third gene selected for further analysis was *BMP8B*, which was down-regulated in UC and up-regulated in CD according to our bioinformatics analysis. Experimental results show a down-regulation of *BMP8B* in both CD and UC groups, more in UC than in CD, confirming the trend of our bioinformatics analysis. The expression of *BMP8B* was significantly down-regulated when UC was compared to CD. The further division of samples based on the location revealed the same pattern, the expression of *BMP8B* was down-regulated in both colon and ileum, more in UC than in CD. When comparing the different disease states, we discovered that the expression of *BMP8B* was down-regulated when UC was compared to CD in the colon and significantly up-regulated when CD in the ileum was compared to CD in the colon. The expression of *BMP8B* could distinguish between CD in the colon or ileum.

The left-right determination factor (LEFTY) and bone morphogenetic protein 8b (BMP8B) are part of the TGF-β superfamily and influence TGF-β signaling which has an important role in maintaining mucosal immunity homeostasis in the bowel [33] and has been shown to be dysregulated in IBD [34]. Therefore, TGF-β signaling has been suggested as a potential therapeutic target in IBD. Recently, a phase II clinical trial has been published showing promising results in achieving remission in active CD following treatment with an anti-SMAD7 oligonucleotide that restores the normal TGF-β signaling [35]. Furthermore, TGF-β is a strong inducer of epithelial-mesenchymal transition [36], which has been implicated in the pathogenesis of fibrosis [37,38] and fistulas [39] in CD. Moreover, LEFTY serves as a repressor of TGF-β signaling by inhibiting Smad2 phosphorylation after activation of the TGF-β receptor and further suppresses downstream events after R-Smad phosphorylation, including the heterodimerization of R-Smads with Smad4 and the nuclear translocation of the R-Smad-Smad4 complex [40].

The *LEFTY1* and *BMP8B* genes have not been associated with IBD yet. Our bioinformatics study shows down-regulation of gene *LEFTY1* and up-regulation of *BMP8B* in CD tissue and up-regulation of *LEFTY1* and down-regulation of *BMP8B* in UC. In addition, the expression of gene *LEFTY2* (logFC = −0.70) decreased, while the expression of gene *BMP8A* increased (logFC = 0.57) in CD tissue, but not in UC. Our experimental results show down-regulation of gene *BMP8B* in both, CD and UC tissues, and down-regulation of *BMP8B* when UC was compared to CD, confirming the trend of bioinformatics analysis. Experimentally, *LEFTY1* was slightly up-regulated in CD and slightly down-regulated in UC, although the differences were not significant.

While the *BMP8B* expression in the colon is unknown, an independent study showed an expression of *LEFTY1* in the colon. The *LEFTY1* mRNA was present in basal cells of the crypts, whereas LEFTY1 protein was localized in the apical region [41]. The gene *LEFTY1* increased in various tissues and in cancer. Miyata et al. reported that *LEFTY* expression is induced by TGF-β in several pancreatic cancer cell lines, all of which contained a RAS mutation [42]. It was previously associated with ovarian epithelial carcinoma through up-regulation of expression at the mRNA and protein levels [43]. The *BMP8B* is also known as a tumor suppressor in cancer of the stomach [44,45], ovary [46] and pancreas [47]. Moreover, *BMP8* has been mostly associated with male reproductive system development [48].

## 5. Conclusions

By analyzing the gene expression profiles of four UC and three CD associated GEO projects, we found differences in the expression profiles between UC and CD for three cytokines, i.e., *INSL5*, *LEFTY1* and *BMP8B*, which we experimentally validated. Their expression profiles were analyzed in CD, UC and normal samples of the colon and ileum. Gene *INSL5* was expressed only in the colon, while *BMP8B* and *LEFTY1* have the potential to distinguish between UC and CD in the colon or ileum.

Furthermore, the expression profiles of *INSL5*, *LEFTY1* and *BMP8B* seem promising, warranting further studies, such as DNA methylation and protein expression to provide more evidence of their potential diagnostic significance in CD and UC.

## Figures and Tables

**Table 1:** Number of samples used in bioinformatics analysis. UC, ulcerative colitis; CD, Crohn’s disease; NA, not applicable.

GEO Project	Samples	Normal	UC	CD	Platform
GSE13367	17	9	8	NA	Affymetrix Huma Genome U133 Plus 2.0 Array
GSE48958	15	8	7	NA	Affymetrix Human Gene 1.0 ST Array
GSE53306	28	12	16	NA	Illumina HumanHT-12 WG-DASL V4.0 R2
GSE65114	28	12	16	NA	Affymetrix Human Gene 2.0 ST Array
GSE24287	72	25	NA	47	Agilent Whole Human Genome Microarray 4x44K G4112F
GSE66207	33	13	NA	20	Illumina HiSeq 2500
GSE100833	327	168	NA	159	Affymetrix HT HG-U133+ PM Array Plate

**Table 2:** Clinical characteristics of patients. n, number of samples; F, female; M, male.

	n	F/M	Average Age
Crohn’s disease	27	14/13	42 ± 19
Colon	13	8/5	43 ± 19
Ileum	14	6/8	42 ± 19
Ulcerative colitis			
Colon	15	9/6	49 ± 17
Normal mucosa	22	17/5	44 ± 22
Colon	10	8/2	44 ± 24
Ileum	12	9/3	44 ± 22

**Table 3:** TaqMan Gene Expression Assays used in experiment. bp, base pairs.

Gene	Assay ID	Product (bp)
*B2M*	Hs99999907_m1	75
*IPO8*	Hs00183533_m1	71
*BMP8B*	Hs01629120_s1	105
*INSL5*	Hs00193884_m1	119
*LEFTY1*	Hs04235412_sH	109

**Table 4:** Differentially expressed genes with inverse regulation in ulcerative colitis and Crohn’s disease. CD, Crohn’s disease; UC, ulcerative colitis; logFC, logarithm of fold change.

Gene	CD		UC		Difference
	logFC	*p*-Value	logFC	*p*-Value	logFC
*INSL5*	−1.26	0.015	3.22	<0.001	4.48
*VIP*	−1.23	0.003	2.77	<0.001	4.00
*FAM3B*	2.35	0.002	−0.72	0.002	3.07
*NTS*	2.08	0.012	−0.81	0.003	2.89
*LEFTY1*	−1.38	0.043	0.92	0.001	2.30
*SEMA6D*	0.83	0.005	−0.77	0.013	1.60
*FGF2*	−0.66	0.010	0.90	0.003	1.56
*SEMA3A*	−0.66	0.022	0.82	<0.001	1.48
*EREG*	−0.62	0.047	0.81	0.004	1.43
*BMP8B*	0.57	0.017	−0.79	0.007	1.36

**Table 5:** The union of functional enrichment analysis of cytokines differentially expressed in ulcerative colitis and Crohn’s disease. UC, ulcerative colitis; CD, Crohn’s disease; NA, not applicable; *, number of significant categories.

Category	Number of Available Categories	UC *	CD *	Union *
Gene ontology (GO)				
Biological processes	6168	1322	100	1323
Cellular component	758	4	NA	4
Molecular functions	1550	41	14	41
Pathway analysis				
BioCarta pathways	310	22	NA	22
KEGG pathways	280	61	6	61
Reactome pathways	1300	47	2	47
Phenotype				
NIA-Phenotypes	704	129	NA	129

## Data Availability

All data used in this paper are available in the article and Supplementary Materials.

## Supplementary Materials

The following are available online at https://www.mdpi.com/article/10.3390/genes12101477/s1, Table S1: List of Cytokine-coding genes, Table S2: List of significantly expressed genes and probes in Crohn’s disease or ulcerative colitis, Table S3: List of significant Gene Ontology terms, pathways and phenotypes for genes significantly expressed in Crohn’s disease and ulcerative colitis.
